# Nailfold Capillaroscopy Patterns Are Associated with Impairment of DLCO in Very Early Diagnosis of Systemic Sclerosis: Insights of a Pulmonary-Vascular Interplay

**DOI:** 10.3390/diagnostics16172780

**Published:** 2026-08-29

**Authors:** Eugenio Capparelli, Barbara Ruaro, Eleonora Zaccara, Marco Vicenzi, Greta Pellegrino, Daniela Bompane, Laura Castelnovo, Antonio Tamburello, Elisabetta Ricchiuti, Paolo Carlucci, Daniele Colombo, Giorgio Bonardi, Maria Sole Chimenti, Antonino Mazzone, Paola Maria Luigia Faggioli

**Affiliations:** 1 Rheumatology Unit, Azienda Socio-Sanitaria Territoriale Ovest Milanese, Legnano Hospital, Legnano, 20025 Milan, Italy; eleonora.zaccara@asst-ovestmi.it (E.Z.); daniela.bompane@asst-ovestmi.it (D.B.); laura.castelnovo@asst-ovestmi.it (L.C.); antonio.tamburello@asst-ovestmi.it (A.T.); paola.faggioli@asst-ovestmi.it (P.M.L.F.); 2 Rheumatology, Allergology and Clinical Immunology, Department of Systems Medicine, University of Rome Tor Vergata, 00133 Rome, Italy; maria.sole.chimenti@uniroma2.it; 3 Department of Pulmonology, Cattinara Hospital, University of Trieste, 34139 Trieste, Italy; barbara.ruaro@yahoo.it; 4 Department of Clinical Sciences and Community Health, University of Milan, 20122 Milan, Italy; ma.vicenzi@asst-rhodense.it; 5 Cardiology Unit, Azienda Socio-Sanitaria Territoriale Rhodense, Garbagnate Milanese Hospital, Garbagnate Mialese, 20024 Milan, Italy; 6 IRCCS Ospedale Galeazzi-Sant’Ambrogio, 20152 Milan, Italy; greta.pellegrino@unimi.it; 7 Department of Biomedical and Clinical Sciences, University of Milan, 20157 Milan, Italy; 8 Pneumology Unit, Azienda Socio-Sanitaria Territoriale Ovest Milanese, Legnano Hospital, Legnano, 20025 Milan, Italy; elisabetta.ricchiuti@asst-ovestmi.it (E.R.); paolo.carlucci@asst-ovestmi.it (P.C.); daniele.colombo@asst-ovestmi.it (D.C.); giorgio.bonardi@asst-ovestmi.it (G.B.); 9 Internal Medicine Unit, Azienda Socio-Sanitaria Territoriale Ovest Milanese, Legnano Hospital, Legnano, 20025 Milan, Italy; antonino.mazzone@asst-ovestmi.it

**Keywords:** Systemic Sclerosis, VEDOSS, DLCO, pulmonary function, capillaroscopy

## Abstract

**Background/Objectives**: Data regarding the association between diffusing lung capacity for carbon monoxide (DLCO) and nail-fold Videocapillaroscopy (NVC) patterns are sparse in Very Early Diagnosis of Systemic Sclerosis (SSc; VEDOSS). The aim of this study was to detect differences in VEDOSS patients exhibiting reduced versus normal values of DLCO/single-breath (SB)% predicted (with a cut-off of 80%). **Methods**: A cross-sectional study was conducted in 65 VEDOSS patients, enrolled due to their fulfillment of the 2021 criteria, while not meeting the 2013 ACR/EULAR Criteria. Pulmonary function tests and NVC evaluations were conducted from January to March 2026. Univariate tests were used for intergroup analysis. Logistic and linear regression models were employed to identify predictors of DLCO/SB < 80% and DLCO/SB% values, respectively. *p*-values < 0.05 were considered statistically significant. **Results**: Forty-two (64.6%) patients reported preserved DLCO/SB% and twenty-three (35.4%) exhibited reduced DLCO/SB%. Patients with only ANA positivity were more prevalent in the DLCO-preserved group (*p* = 0.026), while SSc-specific autoantibodies were more frequently present in the DLCO < 80% group (*p* = 0.028), as well as gastrointestinal symptoms (*p* = 0.039). The late pattern was identified exclusively in the DLCO < 80% group (*p* = 0.013), while patients with advanced NVC abnormalities (namely, the active/late pattern) reported lower values of DLCO/SB%. A logistic regression model confirmed a trend toward significance in predicting reduced DLCO for the active/late pattern, while the latter was negatively associated with the unitary increase in DLCO/SB% values (standardized β= −0.38, *p* = 0.008) in linear regression analysis. **Conclusions**: Reduced DLCO/SB (<80%) is associated with an active/late pattern on NVC in VEDOSS.

## 1. Introduction

Systemic sclerosis (SSc) is a rare connective tissue disease (CTD) characterized by a heterogeneous clinical presentation, ranging from indolent lifelong disease to rapidly progressive forms [1]. Shaping disease trajectories and establishing the correct diagnosis can be challenging in the absence of overt skin fibrosis, telangiectasias, calcinosis, or major organ involvement [2]. In the early phases, the hallmark of SSc pathogenesis is represented by distal vasculopathy, which clinically manifests as Raynaud’s Phenomenon (RP). RP is a three-phase event characterized by ischemic vasospasm, cyanosis and reactive hyperemia [3]. Additional features of SSc vasculopathy are puffy fingers, caused by extensive capillary leakage and subcutaneous edema accumulation often preceding sclerodactyly, as well as digital ulcers and fingertip pitting scars, which are painful consequences of prolonged digital ischemia [4,5].

Beyond the functional impairment of the microvascular system, several structural changes in the capillary bed can be detected by nailfold Videocapillaroscopy (NVC) [6]. NVC is a standardized and dynamic tool employed by rheumatologists to uncover the presence of scleroderma-related abnormalities, which can be classified as suggested by Cutolo into early, active and late patterns [7,8].

In recent years, Very Early Diagnosis of Systemic Sclerosis (VEDOSS) preliminary criteria, first proposed in 2011, have been validated [9,10]. The entry criterion is defined by RP, along with the mutual or combined presence of puffy fingers and antinuclear antibodies (ANA). At the VEDOSS stage, due to the scarcity of clinical clues, the detection of SSc-specific autoantibodies (anti-centromere [ACA] and anti-DNA topoisomerase I [anti-Scl70]) and NVC abnormalities represent the few instruments that assist physicians in the identification of patients at risk of progression at five years [10,11].

In the VEDOSS project, the authors failed to apply scleroderma-specific pattern categorization in their risk analysis, thereby defining as “abnormal” NVC images the presence of any giant capillary and/or any degree of capillary loss (from rare to severe), with or without microhemorrhages [10]. However, in a cohort of 102 undifferentiated CTD (UCTD) patients at risk of developing SSc, Riccardi A. et al. detected the presence of avascular areas in three cases, as well as reduced DLCO below 80% in 33 out of 102 subjects [12].

In SSc, NVC abnormalities are often irreversible, and a growing body of literature has highlighted that advanced alterations are predictive of disease evolution toward major organ involvement. For instance, the late NVC pattern, characterized by progressive capillary loss and avascular areas, has been associated with the presence and severity of Pulmonary Arterial Hypertension (PAH) and Interstitial Lung Disease (ILD) [13,14,15], whereas reduced Diffuse Lung Capacity for Carbon monoxide (DLCO) at diagnosis is a well-known predictor of inflammatory, fibrotic and vascular changes at the pulmonary level [16,17,18]. Accordingly, our previous evidence demonstrated that reduced DLCO/single-breath (SB) below 70% represented an additional risk factor for progression in VEDOSS patients [19]. Nonetheless, the relationship between pulmonary function and altered microcirculation detected on NVC has not yet been considered in this population.

Moreover, established risk factors for progression, as proposed by EUSTAR, have focused on the dichotomous presence/absence of any NVC abnormalities, failing to consider whether VEDOSS patients can exhibit specific NVC patterns as per Cutolo’s classification.

Therefore, the present study aims to investigate capillaroscopy differences and pattern distribution in VEDOSS, by comparing patients with preserved DLCO/SB% (≥80% predicted) versus reduced DLCO/SB% (<80%) and to identify predictors of reduced DLCO/SB% in this population.

## 2. Materials and Methods

### 2.1. Study Design and Population

We conducted a cross-sectional observational study including patients followed up regularly at the Scleroderma Unit of Legnano Hospital of Azienda Socio Sanitaria Territoriale Ovest Milanese (Milan, Italy). Enrollment occurred between January and March 2026. Inclusion criteria encompassed (1) an age of 18 years or older, (2) fulfillment of the 2021 validated criteria for VEDOSS diagnosis [10], and (3) the availability of pulmonary function tests and NVC assessment within the enrollment period. Exclusion criteria included: (1) a prior diagnosis of definite SSc according to the 1980 American College of Rheumatology (ACR) and/or 2013 ACR/European Alliance of Associations for Rheumatology (EULAR) classification criteria [20,21], (2) fulfillment of any further ACR or EULAR classification criteria for a definite CTD (like overlap syndromes), (3) any further positivity for CTD-specific autoantibodies other than SSc-specific ones (including ACA, Scl70 and anti-RNA polymerase III), and (4) concurrent pulmonary disease unrelated to SSc—such as chronic obstructive pulmonary disease, asthma, or lung cancer.

All participants provided written informed consent to participate in the study, which was conducted in accordance with the Declaration of Helsinki. Ethical approval was obtained from the local ethics committee (Milan Area 3) under protocol number “6450_17.12.2025_N_bis”.

### 2.2. Clinical and Demographic Data

Demographic variables, including age, sex, and body mass index (BMI), were collected at enrollment. Disease-related clinical features included the presence of RP, puffy fingers, duration of RP and duration of VEDOSS, along with gastrointestinal (GI) involvement (both upper and lower). Data on the autoantibody profile were retrieved from medical records and included ANA, SSc-specific antibodies (ACA, Scl70 or anti-RNA polymerase III) and ENA assessments (RoSSA, LaSSB, JO1, U1-snRNP, Sm).

### 2.3. Pulmonary Function Tests

Pulmonary function tests (PFTs) were performed using global spirometry for volume measurement and the single-breath technique for carbon monoxide diffusion according to American Thoracic Society (ATS) guidelines [22]. PFTs were conducted employing body plethysmography (MasterScreen Body, Jaeger Medical GmbH, Höchberg, Bavaria, Germany) by two trained respiratory technicians. At least two reproducible and technically acceptable tests were required for each parameter [22]. PFT parameters were further interpreted by two certified pulmonologists (G.B. and E.R.) blinded to patients’ clinical data, following the 2022 ATS/ERS technical standard on interpretative strategies [23].

The primary parameter of interest was DLCO single-breath (SB), expressed as a percentage of predicted values (% predicted), corrected for BMI, hemoglobin, age and ethnicity. A DLCO value ≥ 80% was considered normal, whereas a value <80% was classified as reduced. The present cut-off was employed based on clinical decision and previous evidence on its potential to discriminate functional impairment [12,24]. Forced vital capacity (FVC) and the FVC/DLCO ratio were also recorded.

### 2.4. ILD or PAH Exclusion

As no evidence-based recommendations or guidelines support systematic high-resolution computed tomography (HRCT) execution for ILD detection in VEDOSS patients, previous radiology reports (within the year prior to recruitment) on chest imaging (either HRCT or X-ray) were employed to confirm the absence of ILD [25]. Moreover, the DETECT algorithm was used to estimate the likelihood of PAH, with no patients reaching the threshold to undergo right heart catheterization (RHC) [26]. Data on echocardiographic parameters were recorded from the last available assessment and included Tricuspidal Annular Plane Systolic Excursion (TAPSE) and systolic Pulmonary Arterial Pressure (sPAP).

### 2.5. NVC Evaluation

NVC evaluations were performed by experienced rheumatologists (E.Z. and P.M.L.F.) using a videocapillaroscope at 200× magnification. NVC examination was conducted following a 20 min rest period in a temperature-controlled environment. Clinical reporting followed national guidelines to ensure a reliable and reproducible assessment [8]. NVC-related patterns were classified according to Cutolo’s criteria and categorized as early (few giant capillaries, preserved capillary distribution and absence of evident capillary loss), active (frequent giant capillaries and microhemorrhages, moderate capillary loss and architectural disorganization) and late (irregularly enlarged capillaries, few or absent giant capillaries, absence of microhemorrhages, severe capillary loss with avascular areas, marked architectural disorganization and frequent ramified capillaries, indicative of neoangiogenesis) [7]. Moreover, the non-specific (non-scleroderma) pattern indicates the absence of capillaroscopic criteria for scleroderma microangiopathy [27].

### 2.6. Statistical Analysis

Results from the study were expressed in their aggregate form. Categorical variables were reported as counts and percentages, while continuous ones were expressed as the mean ± standard deviation (SD) or median and 25th and 75th interquartile range (IQR_1–3_), depending on data distribution. The Shapiro–Wilk test was used to assess the normal distribution of continuous variables. Intergroup comparisons between DLCO ≥ 80% and DLCO < 80% were performed using independent *t*-tests for normally distributed continuous variables and Mann–Whitney U tests for non-normal distributions. Chi-square and Fisher’s exact tests were used for categorical variables. Fisher’s exact test was used when the expected number of cases in each cell was less than five. Since the analysis involved a 2 × 2 contingency table, no post hoc test was performed.

Multivariable analysis was employed twice. First, we conducted a binary logistic regression to identify determinants of reduced DLCO < 80% (outcome), incorporating as independent variables of interest SSc-specific autoantibody positivity (ACA and Scl70) and the active/late NVC pattern, as per the main purpose of the study. Odds ratios (OR) and 95% confidence intervals (CI) were accordingly reported. Second, a linear regression model was constructed to assess the independent effect of the active/late NVC pattern on DLCO/SB% values (continuous variable), after adjusting for clinically relevant covariates such as age, BMI, and SSc-specific antibodies. We applied multivariable models using the 1:10 rule of thumb, meaning that we included one variable in the multivariable analysis for every ten events of DLCO below the established cut-off.

For these analyses, statistical significance was set at a *p*-value less than 0.05. All analyses were conducted using SPSS version 29.0 (IBM, Armonk, NY, USA).

## 3. Results

### 3.1. Main Characteristics of the Entire Cohort

A total of 65 patients in a stable VEDOSS condition were recruited. There was a marked predominance of female patients (61/65, 93.8%) compared to males (4/65, 6.2%) and an average BMI of 23.4 ± 6.3 kg/m^2^. The mean age at enrollment was 58.2 ± 15.3 years, the mean age at VEDOSS diagnosis was 47.9 ± 14.8 years, and the mean age at RP onset was 41.2 ± 17.6 years. The median durations of VEDOSS and RP were 9 years (IQR_1–3_ 5–13.75) and 13 years (IQR_1–3_ 8–24), respectively. The percentages of patients with an RP and VEDOSS duration exceeding 10 years were 52.3% and 46.2%, respectively. A total of 26 out of 65 patients (40%) were positive exclusively for ANA, while 19 (29.2%) were ANA-negative, and 20 (30.8%) had SSc-specific autoantibodies (ACA. Scl70). No patients exhibited further specific positivity indicating potential overlaps with other connective tissue diseases. Gastrointestinal symptoms were reported in 52.3% of cases.

A scleroderma pattern was predominant in the whole cohort, reaching 92.3% compared to 7.7% for the non-scleroderma pattern. Among the former, the early pattern was the most frequent (37/65, 56.9%), followed by the active pattern (19/65, 29.2%) and the late pattern (4/65, 6.2%).

The median DLCO/SB % pred. of the population was 85% (IQR_1–3_ 75–92), the median FVC% pred. was 105% (IQR_1–3_ 93.5–120) while the median FVC/DLCO ratio was 1.21 (IQR_1–3_ 1.09–1.52). A total of 42 patients (64.6%) had preserved DLCO/SB% pred. values (≥80%) and 23 (35.4%) had DLCO < 80%. Only two patients exhibited an FVC% pred. less than 80% and one less than 70%, while an FVC/DLCO ratio greater than 1.5 was found in eighteen cases.

Regarding treatment, iloprost was the most frequently administered vasodilating therapy (48 patients, 73.8%), followed by low-dose aspirin (LDA) (46 patients, 70.7%). Calcium channel blockers (CCBs) were used in 28 patients (43.1%), while hydroxychloroquine (HCQ) was prescribed in 22 patients (33.8%).

Moreover, 40 out of 65 patients underwent chest HRCT, and no signs of ILD were detected. The remaining group conversely underwent chest X-ray with no suspicion of ILD. Echocardiographic parameters revealed that patients with reduced DLCO exhibited lower TAPSE values of 20.8 ± 3.1 mm compared to the DLCO-preserved group (22.3 ± 3.2 mm, *p* = 0.120), and a higher mean sPAP of 27.3 ± 5.4 vs. 26.3 ± 6.5 mmHg (*p* = 0.695).

### 3.2. Comparative Analysis Between Patients with Reduced Versus Preserved DLCO

When comparing patients with preserved DLCO (N = 42) versus reduced DLCO (N = 23), an equal distribution of sex was found [Table 1]. The groups did not differ according to the age at enrollment, RP onset, or VEDOSS diagnosis, along with VEDOSS and RP duration. However, a significant difference was found in the percentage of patients reporting a VEDOSS duration greater than 10 years in the DLCO-reduced group (*p* = 0.022), as well as RP duration (*p* = 0.039). Patients with preserved DLCO values tested more frequently positive for ANA alone (*p* = 0.026), while the DLCO-reduced group more frequently exhibited SSc-specific antibody positivity (*p* = 0.028), the same group reported more frequently gastrointestinal symptoms (*p* = 0.039). Treatment exposure to iloprost, CCBs, LDA and HCQ was comparable between the groups.

**Figure 1:**
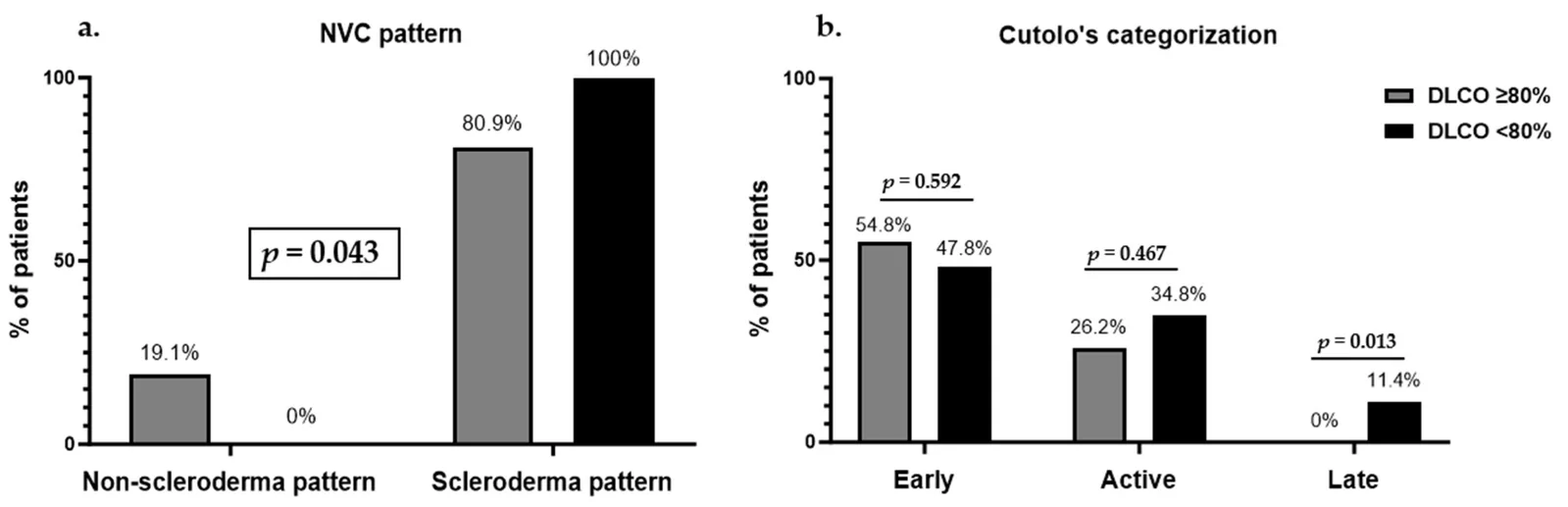
Bar chart of NVC findings according to Cutolo Classification by DLCO/SB% pred. value. (**a**). shows NVC scleroderma apttern distribution between the two groups. (**b**). shows NVC pattern distribution according to Cutolo’s categorization between the two groups. Abbreviations. DLCO = Diffusing capacity for carbon monoxide, NVC = Nailfold Videocapillaroscopy.

Regarding NVC evaluation, the total cohort with reduced DLCO reported a scleroderma pattern, while the DLCO-preserved group reported a scleroderma pattern in 80.9% (34 out of 42) of cases and a non-scleroderma pattern in 19.1% (8 out of 42) (*p* = 0.043) [Figure 1a and Supplementary Table S1]. The late pattern was exclusively observed in the DLCO < 80% cohort (*p* = 0.013) [Figure 1b]. Early and active patterns were distributed among both groups without a significant difference, with the early pattern found in 23/42 (54.8%) and the active pattern in 11/42 (26.2%) in the DLCO-preserved cohort. Conversely, in the DLCO-reduced cohort, the distribution was as follows: early in 11/23 (47.8%), active in 8/23 (34.8%), and the late pattern in 4/23 (17.4%) [Figure 1b].

FVC values did not differ significantly between the groups (*p* = 0.67). However, FVC/DLCO ratios were higher in the DLCO-reduced group.

### 3.3. Comparative Analysis of PFTs According to NVC Findings

We conducted a comparison of PFT results according to NVC findings by dividing the population into two groups: early pattern/non-specific NVC abnormalities (n = 42) vs. patients showing an active/late pattern (n = 23) [Supplementary Table S2].

No significant differences were observed in lung volumes between the two groups (mean FVC values of 108 ± 21.8% vs. 107.7 ± 19.9%, *p* = 0.958). In contrast, lower DLCO/SB % values were found in the active/late pattern group compared with the early/non-specific group (78.9 ± 12.3% vs. 86.5 ± 13.2%, *p* = 0.025). However, when corrected for alveolar volume, DLCO/AV values were similar between the groups (86.7 ± 19.5% vs. 86.5 ± 15.3%, *p* = 0.975). The FVC/DLCO ratio tended to be higher in the active/late pattern group (1.4 ± 0.4 vs. 1.2 ± 0.3, *p* = 0.086).

### 3.4. Multivariable Analysis

The overall multivariable linear regression model was statistically significant (F(4,45) = 3.066, *p* = 0.026), explaining 21.4% of the variance in DLCO/SB % predicted (R^2^ = 0.214; adjusted R^2^ = 0.144). After adjustment for the covariates included in the model (age, BMI and SSc-specific antibodies), the presence of an active/late NVC pattern was independently associated with a lower DLCO/SB % predicted (β standardized = −0.38, 95% CI −17.95 to −2.79, *p* = 0.008), corresponding to an approximately 10-point lower DLCO/SB % predicted compared with patients without an active/late NVC pattern [Table 2]. In the logistic regression analysis with DLCO < 80% as the dependent variable, neither the presence of SSc-specific autoantibodies (ACA, SCl70 and RNA-pol. III) nor an active/late NVC pattern was significantly associated with the outcome. Specifically, SSc-related autoantibodies showed an adjusted OR of 1.87 (95% CI: 0.62–5.63, *p* = 0.266), while an active/late NVC pattern showed a higher OR of 2.72 with a trend toward significance (95% CI: 0.92–8.03, *p* = 0.071).

## 4. Discussion

The present cross-sectional study aimed to investigate the interplay between microvascular injury and lung function, measured by DLCO, in a monocentric cohort of VEDOSS patients. We observed that patients exhibiting both active and late patterns at the nail-fold were more likely to report lower values of DLCO/SB% compared with patients with early capillaroscopic abnormalities. While the active pattern was relatively evenly distributed between the two groups (34% in the DLCO-reduced group vs. 26% in the DLCO- preserved group), the late pattern was exclusively detected in patients with reduced DLCO. Moreover, the multivariable model confirmed the negative linear association between DLCO/SB% values and the active/late pattern, while a trend toward association was noticed in the binary logistic regression employing the cut-off of 80% to define patients with reduced DLCO.

PFTs are essential to track the prognosis of cardiopulmonary complications in SSc [24,28]. Importantly, both FVC and DLCO reduction at baseline have been found useful in predicting progression and mortality in SSc-associated ILD [29,30]. Nonetheless, as up to two-thirds of SSc-ILD patients initially exhibit normal PFTs, several concerns have arisen regarding their capacity to predict ILD onset [31]. Bernstein E. et al. found that DLCO below 80% had better sensitivity compared with FVC and Total Lung Capacity (TLC) in detecting ILD on HRCT in patients with early diffuse cutaneous SSc, and as our previous evidence proposed DLCO/SB% pred. below 70% as a potential marker of progression in VEDOSS, we accordingly decided to assess functional lung impairment mainly by employing DLCO [32].

DLCO is also a key parameter assessed in patients with Pulmonary Hypertension, as its reduction in idiopathic PH is correlated with poor survival and a diminished response to treatment, while in SSc, it allows the stratification of suspected cases of PAH [33,34,35].

DLCO describes gas diffusion through the alveolar–capillary interface, suggesting that its reduction could be explained, at least in part, by the widespread microangiopathy typical of SSc pathogenesis [36,37]. Generally, a decrease in DLCO can occur as a result of several factors, including thickening of the alveolar–capillary membrane due to the early alveolar inflammatory and fibrosis-driven remodeling processes [38]. Thus, a thickened interstitium, remodeling of the lung vasculature and a reduction in capillary surface area induce ventilation–perfusion imbalance, which becomes irreversible in advanced stages [38]. Therefore, DLCO assessment might potentially inform physicians about both early vascular and alveolar epithelium derangement [39]. Our evidence, demonstrating a more prominent reduction in DLCO (35.4%) compared to the FVC decrease seen in only two patients with VEDOSS, aligns with this pathophysiological proof, suggesting the presence of initial pulmonary vascular derangement even in the absence of detectable ILD or PAH.

One of our most relevant findings is the exclusive presence of the late pattern among patients with reduced DLCO, supporting the concept that structural microvascular derangement parallels early gas exchange deterioration in the lungs. Accordingly, we demonstrated that capillaroscopic patterns, rather than the mere presence of any NVC abnormalities, appear more clinically meaningful in very early disease. However, the logistic regression analysis did not demonstrate a statistically significant association with the dichotomous outcome of DLCO below 80%. This lack of statistical significance is attributable to the small sample size and the limited number of patients exhibiting a late pattern, which was limited to the DLCO-reduced population. In contrast, the linear regression model, which considered DLCO as a continuous variable, revealed a significant independent association between active/late patterns and lower gas exchange values. This finding underscores that dichotomization may lead to a loss of information regarding the prediction of lung function deterioration.

Therefore, since their validation, the VEDOSS criteria have opened a new window of opportunity for prognostication and early intervention in SSc. The VEDOSS state substantially mirrors preclinical rheumatoid arthritis, where risk factors for progression, such as anticitrullinated peptide antibody positivity with inflammatory arthralgias, and preventive therapeutic strategies have been established [40]. Nowadays, in VEDOSS, no disease-modifying drugs have proved effective in halting the transition toward definite SSc, and due to the heightened risk of progression and subtle organ involvement (early cardiac and gastrointestinal manifestations), identifying patients at higher risk seems to be of paramount importance [41,42,43,44].

The higher prevalence of gastrointestinal symptoms in patients with reduced DLCO mirrors the previous evidence of the EUSTAR group, in which an increased risk of esophageal dysmotility was found in patients with very early disease [44]. Interestingly, while SSc-specific autoantibodies remain important markers of disease transition, their role in predicting gas exchange dysfunction may be less direct compared with microvascular damage, as they were not associated with DLCO reduction in the multivariable analysis. The present result mirrors the evidence of the low influence of SSc-specific antibodies on PAH severity in established SSc [45]. Furthermore, no patients in our cohort achieved the threshold for RHC using the DETECT algorithm, nor were differences between the two groups found for echocardiographic-derived parameters, suggesting that echocardiography at early stages does not aid in patient stratification.

Several limitations should be acknowledged. First, the cross-sectional design precludes any inference of causality. Longitudinal studies are needed to determine whether NVC changes precede or parallel DLCO decline. Second, the relatively small sample size may have limited the statistical power of our analyses. Third, although efforts were made to exclude overt pulmonary disease, subclinical alterations detectable by HRCT may have been missed.

Additionally, treatment exposure was comparable between the groups, but its potential impact on microvascular and pulmonary parameters cannot be fully excluded. Specifically, the high prevalence of iloprost use reflects the treatment strategy routinely adopted at our center. The availability of a dedicated outpatient infusion service and favorable local organizational conditions facilitate regular iloprost administration compared with CCB prescription. Therefore, this prescribing pattern should be interpreted as representative of local clinical practice rather than a broadly applicable therapeutic approach and could have had a potential influence on the findings. Future studies should explore the role of vasodilating/vasoactive therapies and immunosuppressants in modulating the relationship between lung function and vasculopathy in very early SSc. Finally, although NVC assessment was performed by experienced operators following standardized procedures, some degree of interobserver variability should be considered.

## 5. Conclusions

From a clinical perspective, our results highlight the potential usefulness of NVC pattern stratification in the risk assessment of VEDOSS patients. The combination of an active/late NVC pattern and reduced DLCO/SB may identify patients who warrant closer cardiopulmonary assessment and more stringent follow-up, for instance, requiring echocardiographic or HRCT assessments more frequently compared with patients with an indolent respiratory and capillaroscopic profile. This approach could contribute to the earlier recognition of patients at higher risk of progression, although prospective longitudinal studies are needed to determine whether such stratification translates into clinically meaningful benefits.

## Figures and Tables

**Table 1:** Comparative univariate analysis between groups according to DLCO/SB values.

	DLCO ≥ 80% n = 42	DLCO < 80% N = 23	*p*-Value
Female, n (%)	38 (90.5)	23 (100)	0.288
Male, n (%)	4 (9.5)	0	
Age at enrollment, years, mean ± S.D.	56.5 ± 15.9	61.2 ± 13.8	0.237
Age At VEDOSS Diagnosis, years, mean ± S.D.	46.9 ± 15.4	49.9 ± 13.8	0.439
Age At RP onset, years, mean ± S.D.	40.5 ± 17.7	41.4 ± 17.7	0.845
Duration of VEDOSS, years, mean ± S.D.	9.7 ± 7.8	11.8 ± 7.4	0.295
Duration of RP, years, mean ± S.D.	16.7 ± 11.9	17.0 ± 10.3	0.919
Body Mass Index, (Kg/m^2^), mean ± S.D.	23.4 ± 6.6	23.3 ± 6.0	1.0
RP > 10 Years, n (%)	18 (42.8)	16 (69.5)	0.039
VEDOSS > 10 Years, n (%)	15 (35.7)	15 (65.2)	0.022
Puffy Hands, n (%)	9 (21.4)	7 (30.4)	0.420
ANA (positive) only, n (%)	21 (50)	5 (21.7)	0.026
ANA (negative), n (%)	12 (28.6)	7 (30.4)	0.875
SSc Specific Autoantibodies (positive), n (%)	9 (21.4)	11 (47.8)	0.028
Gastrointestinal symptoms, n (%)	18 (42.9)	16 (69.6)	0.039
**Treatment Exposure, n (%)**			
Iloprost iv	32 (76.2)	16 (69.6)	0.561
Calcium Channel Blockers	19 (45.2)	9 (39.1)	0.634
Low-Dose Aspirin	32 (76.2)	14 (60.9)	0.194
Hydroxychloroquine	15 (35.7)	7 (30.4)	0.667

Abbreviations. ANA = antinuclear antibodies, DLCO = Diffusing capacity of the Lungs for Carbone monoxide, N = number, RP = Raynaud’s Phenomenon, S.D. = Standard Deviation, SSc = Systemic Sclerosis, VEDOSS = Very Early Diagnosis of Systemic Sclerosis.

**Table 2:** Linear regression model.

DLCO/SB % Pred. (Dependent Variable)	Beta-Coefficient	Standard Error	Standardized Beta-Coefficient	95% CI	Significance
Constant	98.75	10.39		77.81; 119.69	<0.001
Age	−0.19	0.14	0.21	−0.47; 0.077	0.155
BMI	−0.16	0.34	−0.06	−0.84; 0.52	0.648
SScAb	3.80	3.90	0.14	−4.06; 11.66	0.335
Active/late NVC pattern	−10.37	3.76	−0.38	−17.95; −2.79	0.008

Abbreviations. BMI = Body Mass Index; SScAb = Systemic Sclerosis specific autoantibodies NVC = Nailfold Videocapillaroscopy.

## Data Availability

The data that support the findings of this study are available from the corresponding author upon reasonable request.

## Supplementary Materials

The following supporting information can be downloaded at: https://www.mdpi.com/article/10.3390/diagnostics16172780/s1, Supplementary Table S1: NVC pattern distribution according to Cutolo’s Categorization. Supplementary Table S2: Univariate analysis of PFTs according to NVC findings.

## Abbreviations

The following abbreviations are used in this manuscript:

ACA	Anti-centromere antibodies
ACR/EULAR	American College of Rheumatology/European Alliance of Associations for Rheumatology
ANA	Antinuclear antibodies
ATS	American Thoracic Society
ATS/ERS	American Thoracic Society/European Respiratory Society
BMI	Body Mass Index
CCB	Calcium Channel Blockers
CI	Confidence Interval
CTD	Connective Tissue Disease
DETECT	Detection of Pulmonary Arterial Hypertension in Systemic Sclerosis
DLCO	Diffusing Capacity of the Lung for Carbon Monoxide
EUSTAR	European Scleroderma Trials and Research Group
FVC	Forced Vital Capacity
GI	Gastrointestinal
HCQ	Hydroxychloroquine
HRCT	High-Resolution Computed Tomography
ILD	Interstitial Lung Disease
IQR_1–3_	Interquartile Range (25th–75th percentile)
LDA	Low Dose Aspirin
NVC	Nailfold Videocapillaroscopy
OR	Odds Ratio
PAH	Pulmonary Arterial Hypertension
PFT	Pulmonary Function Tests
PH	Pulmonary Hypertension
Pred.	Predicted
RHC	Right Heart Catheterization
RP	Raynaud’s Phenomenon
SB	Single Breath
Scl70	Anti-topoisomerase I antibodies (Anti-Scl-70 antibodies)
S.D.	Standard Deviation
SSc	Systemic Sclerosis
TLC	Total Lung Caspacity
