# Supplementary material for: Nailfold Capillaroscopy Patterns Are Associated with Impairment of DLCO in Very Early Diagnosis of Systemic Sclerosis: Insights of a Pulmonary-Vascular Interplay
Source: Diagnostics (Basel). 2026 Aug 29;16(17):2780. doi: 10.3390/diagnostics16172780 (PMC13565217; doi:10.3390/diagnostics16172780)
Supplement: Supplementary file 1 [file diagnostics-16-02780-s001.zip › diagnostics-4500700-supplementary.pdf]

Supplementary Table S1. NVC pattern distribution according to Cutolo's Categorization.

NVC PATTERNS	DLCO ≥80% N=42	DLCO<80% N=23	p-values
NON SCLERODERMA	8 (19.1%)	0	**0.043 (Fisher exact test)**
EARLY	23 (52.8%)	11 (47.8%)	**0.592 (chi-square)**
ACTIVE	11 (26.2%)	8 (34.8%)	**0.467 (chi-square**
LATE	0	4 (11.4%)	**0.013 (Fisher exact test)**

Abbreviations. DLCO= Diffuse lung capacity for carbon monoxyde. NVC= Nailfold Videocapillaroscopy

Supplementary Table S2. Univariate analysis of PFTs according to NVC findings.

	Early pattern/non-specific abnormalities	Active/late pattern	p-value
FVC%, mean ± S.D.	108.0 ± 21.8	107.7 ± 19.9	0.958
DLCO/SB%, mean ± S.D.	86.5 ± 13.2	78.9 ± 12.3	**0.025**
DLCO/AV%, mean ± S.D.	86.5 ± 15.3	86.7 ± 19.5	0.975
FVC/DLCO ratio, mean ± S.D.	1.2 ± 0.3	1.4 ± 0.4	0.086

Abbreviations. FVC = Forced Vital Capacity, DLCO = Diffusing Lung capacity of carbon monoxide; SB = Single breath, AV = Alveolar volume.
